# Lev Tolstoy, A Founder of Democratic Education

**DOI:** 10.1007/s12124-024-09860-w

**Published:** 2024-07-22

**Authors:** Eugene Matusov

**Affiliations:** University of Delaware, Newark, DE USA

**Keywords:** Democratic education, Tolstoy, Educational offerings, Progressive education, Educational coercion, Anarchistic education, Naturalism, Self-organizing

## Abstract

In this philosophical-theoretical study of Lev Tolstoy’s pedagogical legacy of his Yasnaya Polyana school in the Russian Empire (1859–1862), I raised three major questions: (1) was Lev Tolstoy a democratic educator, and if so, why can one claim that, (2) if so, what kind of a democratic educator was he, and (3) what kind of limitations to his democratic education have I observe and what were the sources of these limitations? My answer to the first question was unequivocally positive. I argue that Tolstoy was the conceptual founder of democratic non-coercive education and the first known practitioner of democratic education for children. In my view, his democratic education was based on educational offerings provided by the teachers. His democratic educational philosophy was based on non-coercion, naturalism, anarchism, liveliness, pragmatism, pedagogical experimentation, student responses, pedagogical self-reflection, and dialogism. At the same time, his democratic education was limited to his uncritical acceptance of conventionalism. Tolstoy’s attraction to Progressive Education was facilitated by ignoring his enormous powers, both explicit and implicit, that he manifested exercised in the school and enacted through his “pervasive informality.” In my judgment, Tolstoy overemphasized pedagogy over self-education and did not distinguish learning from education. Still, Tolstoy’s pioneering work in democratic education, both in theory and practice, remains mostly unacknowledged and unanalyzed while continuing to be highly relevant and potentially influential.

My central thesis is that Lev Tolstoy^1^ was the first theoretician of democratic education. Also, it is highly possible that he was the first known practitioner of institutional democratic education as applied to children, not adults. In the historical literature, we have clear examples of informal and formal democratic education that existed long before Lev Tolstoy, such as the Socratic Agora, Aristotle’s Lyceum, Plato’s Academy, the Medieval University of Students in Southern and Central Europe, European and North American educational institutions of public lectures and studies for adults, museums, etc. (Shugurova et al., 2022). Studying democratic education, I could not find any theoretician of democratic education or an institutional democratic education practitioner for children before Leo Tolstoy’s writings or his pedagogical innovative practice in the Yasnaya Polyana school that he established in 1859 for peasant children and adults (1859–1962). Actually, he kept his pedagogical experimentations at the school (in broader settings by publishing books for peasant children) and pedagogical writings between 1872, when he reopened the Yasnaya Polyana school (Wilson, 2015), and the late 1870s or early 1880s^2^ (Tolstoy, 1899a, 1967; Tolstoy & Blaisdell, 2000).

Democratic education is a polysemic notion. Elsewhere, I have abstracted at least five distinct understandings of this term (Matusov, 2023b). Here, by democratic education, I mean recognition of the student (the educatee) as the primary decision-maker for their own education: whether to study, what to study, how to study, when and where to study, for what purpose to study, with whom to study, to determine what constitutes the quality of education, and so on. The educatee may make these decisions autonomously (i.e., autodidact), with peers, and/or with a trusted educator.

My scholarship on Tolstoy presented here is philosophical and theoretical rather than historical. My “supertask,” using Stanislavky’s term, was to reintroduce Tolstoy as the first democratic educator, abstract his philosophical ideas, and engage them in the contemporary philosophical debates about democratic education. I use historical writings by Tolstoy and about Tolstoy to reconstruct his unique educational philosophy of democratic education so, contrasting to the philosophy of Progressive Education, which is ideologically hegemonic in our days (Matusov, 2021a). In my essay, I will try to address the following philosophical inquiries:

1. Was Tolstoy a democratic educator in his theoretical ideas and practice at the Yasnaya Polyana school? If so, what was the evidence of that?
2. What kind of democratic educator was he? What democratic education practices and democratic education philosophy did he subscribe to?
3. What were the limitations of Tolstoy’s democratic education? What were the reasons for those limitations?

In my discussion of Tolstoy’s ideas and practices, I often compared them with the relevant ideas and practices described in modern educational literature.

In my study of Tolstoy’s democratic education theory and practice, as well as Tolstoy’s limitations, I read the sources of his pedagogical writings in Russian (Tolstoy, 2006a), and then their translations in English. Sometimes, I was dissatisfied with the English translations and replaced their translation with mine using brackets.

Finally, it is important to recognize that the existing information and evidence about Tolstoy’s pedagogical ideas and practices are incomplete, absent, contradictory, or fragmented at times. In some cases, I had to speculate. Also, obviously, his ideas and practices changed over time – Tolstoy liked to emphasize that – but some of these changes were non-linear, even having a regressive nature sometimes. I left many important aspects aside, like the systematic review of the existing educational literature on Tolstoy’s pedagogy both in Russia and elsewhere, the analysis of the genesis of his pedagogical ideas (e.g., his travel in Europe to observe the most innovative schools and meet with the most innovative educators of his time in Germany, France, and England as well as his work in a British library to study archival information about the history of international schooling), the memoirs of his peasant students, etc.

In addition, as my colleague Bob Hampel suggested, it can be interesting and important to analyze Tolstoy’s fictional and autobiographical writings for the traces of his pedagogical ideas. I dipped a bit into it by reading Viktor Shklovsky’s monumental book (more than 600 pages! ) on Tolstoy’s life, where he briefly commented on Tolstoy’s fictional novel “Cossacks”, on which Tolstoy worked in parallel to the Yasnaya Polyana school and which might influence his pedagogy. But, of course, this is just the tip of the iceberg. This important analysis should be done in future research. To paraphrase Leonardo da Vinci, “Scholarship is never finished, only abandoned.”

## Natural education free of coercion vs. conventional coercion-based schools

In his educational “manifesto,” a program essay entitled “On people’s education,”^3^ Tolstoy argued for education free from coercion^4^. Tolstoy started his essay by observing a major paradox inherent in institutional education in many countries, which arguably exists nowadays as well. People’s education is perceived by people as their existential need when each and “every [person] unconsciously tends toward education.” (Tolstoy, 1967, p. 3) Both the educated class of people and the government strive to provide institutional education, often even free of charge; this conventional institutional education is often felt by current and former students as boring, irrelevant, taxing, stultifying, and oppressive. Another paradox revealed by Tolstoy is why, despite all pedagogical innovations in the Age of Reason, conventional institutional education remains dogmatic even when it tries to teach reason. Tolstoy blamed coercion for both disturbing paradoxes. He argued that coercion is both the birthmark and the cause of all problems of conventional education.

Throughout his essay and elsewhere in his pedagogical writings, Tolstoy compared and contrasted conventional coercion-based institutional education and naturally occurring everyday education free of coercion. In naturally occurring everyday education free of coercion, education often occurs on the educatee’s demand – something is either interesting, problematic, curious, or puzzling for the educatee. Natural education is *authorial*, where the educatee always remains the author of their own education (i.e., self-education) (Matusov, 2011, 2022). Learning content (i.e., curriculum) is often emergent and not fully known to anyone in advance (cf. “learning curriculum,” Lave, 1992, April). The entire education process is often invisible to the educatee – “the unconscious education,” (Tolstoy, 1967, p. 24). At the same time, learning remains addressive for the educatee: it addresses the educatee’s interest, problem, difficulty, puzzlement, curiosity, inquiry, and so on. When this interest, problem, difficulty, puzzlement, curiosity, or inquiry is exhausted for the educatee, or another more important demand emerges, the educatee is ready to move on to something else educational or non-educational.

Similarly, since this naturally occurring education is free of coercion when the educatee asks an educator for help, it is the educatee who measures the sensitivity and the required depth of the guidance by literally moving away from the educator when the guidance has fulfilled its purpose for the educatee or when it becomes insensitive or unhelpful. Natural education has an inherently inbuilt feedback loop. When guidance becomes insensitive or not on demand anymore, the educatee is free to vote literally with their feet and move away from the educator. Thus, in naturally occurring education free from coercion, learning and guidance answer the educatee’s questions, puzzlements, curiosities, problems, and needs. According to Tolstoy, “If education is good, [the students] will be bound to see it as necessary as air” (Cohen, 1981, p. 243), coercion is not needed. This type of coercion-free education, argued Tolstoy, makes education always relevant, sensitive, and on-demand to the educatee within their ever-changing, if not unique, historical, cultural, and local settings in which this natural education emerges. The future is unpredictable and becomes increasingly unpredictable except for one thing: a permanent, constant, and existential need for naturally occurring on-demand education.

In contrast, the conventional coercion-based institutional education is designed by other people – by educators, government, researchers, experts, “more knowledgeable people” (cf. Vygotsky, 1978), or, simply, more powerful people – for the students. The education designers try to assess what their students must learn, why, how, for how long, and so on. They also design a system of educational coercion, forcing the students to attend their educational institutions. The school curriculum – i.e., what the students must study – is usually well-defined, preexists the educational process, and is designed without the designers’ familiarity with actual students. This *teaching curriculum* (cf. Lave, 1992, April) is often deduced by the education designers from academic disciplines, the past and present needs of society, and imagining the societal future – its future needs and demands on its citizens.

Tolstoy criticized the pre-designed teaching curriculum for its inbuilt insensitivity to the students’ subjectivity and unique, ever-changing, historical, cultural, and local settings in which they live and will live. Tolstoy pointed out that academic subjects often lack a consensus about their content or the importance of their content, or if it exists, the consensus is usually temporary. However, Tolstoy argued, which is even more important for education, that the sociohistorical subjectivity that historically engenders and motivates this academic subject content – its importance for humanity – often escapes the students. The societal goals of education, especially value-based, are politically competing with each other for the monopoly on coercion-based institutional education. The societal and personal future of the students becomes increasingly unknown and unpredictable to the education designers.

Because of coercion, the students cannot move away from the teachers, courses, classrooms, and schools – cannot vote with their feet – when the guidance and entire designed education are insensitive to their needs, which breaks a feedback loop that naturally ensures the quality of education. Instead of viewing student disengagement as helpful marks of such a feedback loop, conventional coercion-based institutionalized education sees it as distressing marks and a signal for applying discipline – i.e., coercion.

This makes conventional coercion-based institutionalized education subservient, *agentic* (Milgram, 1974), involving unconditional conformity to the authority’s arbitrary, if not tyrannic, demands, “…the strongest and most injurious [pedagogical and epistemological] violence [in education] practised on the child when he^5^ is asked to comprehend in precisely the same manner that the teacher comprehends it” (Tolstoy, 1967, p. 46). Tolstoy argued that in the case of its institutional success, conventional coercion-based schools transform lovely, authorial young people into obedient, dull zombies (aka “educated subjects,” Fendler, 1998) in “a disciplinary society” (Foucault, 1995):

It is enough to look at one and the same child at home, in the street, or at school: now you see a vivacious, curious child, with a smile in his eyes and on his lips, seeking instruction in everything, as he would seek pleasure, clearly and frequently strongly expressing his thoughts in his own words; now again you see a worn-out, retiring being with an expression of fatigue, terror, and ennui, repeating with the lips only strange words in a strange language, – a being whose soul has, like a snail, retreated into its house. It is enough to look at these two conditions in order to decide which of the two is more advantageous for the child’s development.

That strange psychological condition which I will call the scholastic condition of the soul, and which all of us, unfortunately, know too well, consists in that all the higher faculties, imagination, creativeness, and inventiveness, give way to other semi-animal faculties, which consist in pronouncing sounds independently from any concept, in counting numbers in succession, 1, 2, 3, 4, 5, in perceiving words, without allowing imagination to substitute images for these sounds, in short, in developing a faculty for crushing all higher faculties, so that only those might be evolved which coincide with the scholastic condition of fear, and of straining memory and attention.

Every pupil is so long an anomaly at school as he has not fallen into the rut of this semi-animal condition. The moment the child has reached that state and has lost all his independence and originality, the moment there appear in him various symptoms of disease – hypocrisy, aimless lying, dullness, and so forth, – he no longer is an anomaly: he has fallen into the rut, and the teacher begins to be satisfied with him. Then there happen those by no means accidental and frequently repeated phenomena, that the dullest boy becomes the best pupil, and the most intelligent the worst (Tolstoy, 1967, pp. 16–17).

However, I sense a gap in Tolstoy’s argumentation: his “elephant in the room.” Why democratic *schooling*? Why *institutionalized* education? Why is naturally occurring everyday education free of coercion not enough? Why did Tolstoy himself become a *designer* of democratic institutionalized education after criticizing other education designers? I could not find a direct answer in his pedagogical writings, but I can imagine his implicit response to these questions.

I suspect that his answer might be historical. Many contemporary societies increasingly require contact among very diverse and alien communities, persons, societies, institutions, practices, and so on. Tolstoy’s interest in education coincided with the abandonment of slavery in the Russian Tsarist Empire (1861). Tolstoy tried to free his peasant slaves^6^ in 1856 but was unsuccessful because his peasants did not trust him^7^. Freed peasants might drastically broaden their social, geographical, political, cultural, and epistemological mobility. Schools could and can provide such mobility.

Another possible answer that Tolstoy might come up with might be students’ existential drive for self-actualization, requiring exposure to diverse academic areas for the students to try to experience themselves. Some peasant parents appreciated their children’s emergent self-actualization at the Yasnaya Polyana School: “Among these boys, who make up the majority, the most joyful phenomenon for us are the boys who were initially [enrolled in the school by their fathers unconsciously as a tribute to the popular fashion] but have come to love learning to such an extent that their fathers now submit to the desires of their children and unconsciously feel that something good is being done with their children, and do not dare to take them out of school” (Tolstoy, 2006b, p. 51). In this democratic institutionalized approach, education is unconscious because it is a byproduct of everyday activities rather than a conscious target, as it is in formal conventional institutionalized education.

I think Tolstoy might justify schooling – schooling without coercion – by the educatee’s need to be prepared for contact with people and settings outside of their immediate surroundings. A somewhat similar justification for schooling was given by John Dewey a few decades later (Dewey, 1915, 1949, 1966). Still, I wonder why Tolstoy thought that the naturally occurring everyday education that promotes learning on-demand could not handle a new contact of the educatee with unfamiliar people as well. It is sufficient to mention that an American democratic educator, John Holt, came to the opposite conclusion of abandoning any institutionalized education in favor of “unschooling” – naturally occurring byproductive learning, which he called “best learning,” (Dickerson, 2019). I believe this conundrum remained unaddressed in Tolstoy’s writings and pedagogical practice in any satisfactory way. However, as we will see below, it was a constant subject of his reflections.

## The Yasnaya Polyana Democratic School of Educational Offerings

Tolstoy argued that his Yasnaya Polyana school emerged organically from the initial chaos, the freedom principle, the teachers’ pedagogical experimentation, and the students’ responses. The principle of freedom for the students and the teachers was based on the Yasnaya Polyana students’ rights not to come to the school or leave the school at any time at their will, to come or leave any classroom at any time, freedom of movement in the classroom, freedom to talk, freedom not engage, etc. and the teachers’ freedom to experiment pedagogically, to expel or limit the students from their lessons, to offer the students their lessons, to teach what and how they wanted to teach, and so on. Unfortunately, Tolstoy did not describe in detail the initial chaos and the transition to the order, which I would describe as “educational offerings” (Cunningham, 2021; Matusov, 2023c):

At first [at the beginning of the Yasnaya Polyana school establishment, ] it was impossible to classify either recitations or the subjects or the recreations or their tasks; everything was in confusion, and all attempts at classification were in vain. At the present time there are students in the first class who themselves insist on following a regular order of exercises, and are indignant when you call them from their lessons, and these scholars are all the time driving away the little ones who disturb them.

In my opinion this external disorder is useful and indispensable, strange as it may seem and inconvenient to the teacher. I shall frequently have occasion to speak of the advantages of this condition of things; of the imaginary inconveniences I will say this: In the first place, this disorder or free order is trying to us, simply because we are accustomed to something entirely different, in which we were [schooled]. In the second place, in this, as in many similar circumstances, the employment of force is due to haste and lack of reverence for human nature. It seems to us that disorder is increasing, becoming more and more violent each instant, that there are no limits to it; it seems to us that there is no other way of putting an end to it than by employing main force, — but really all it requires is to wait a little, and the disorder, or flow of animal spirits, would naturally diminish of itself, and would grow into a far better and more stable order than that which we imagine.

The [school students] — though they are little folk — are nevertheless human beings, having the same requirements as we ourselves, and their thoughts run in the same groove. They all want to learn, and that is the only reason they go to school, and therefore it is perfectly easy for them to reach the conclusion that it is necessary to submit to certain conditions if they would learn anything.

Besides being human beings, they [i.e., the students] form a society of human beings united by one impulse. “And where two or three are gathered together in *My* name there will *I* be also^8^.”

As they are subjected to laws that are simply derived from their own nature, the [school students] do not rebel or grumble; if they were subjected to our old system of interference, they would have no faith in the legality of our ringing bells, regulations, and ordinances (Tolstoy, 1899b, pp. 172–173).

Tolstoy was an educational anarchist (Cohen, 1981), believing that “anarchy is the mother of order”^9^ and that the lawful organic order can only emerge from the chaos, naturalness, freedom, and creative authorship of the participants in the pragmatic responses to the experienced chaos. One can find similar sentiments in many democratic educators such as Neill (1960); Duberman (1969); Matusov, 2023a), and so on. However, knowing the vast diversity of democratic schools established in the XX-XXI centuries, it is unclear to me how much the emerged order in the Yasnaya Polyana school, involving the schedule of lessons, the division on age-based and ability-based classes, using grade marks^10^, teaching well-defined academic subjects such as Mechanical and Comprehensive Reading, Writing, Calligraphy, Grammar, Sacred History, Russian History, Drawing, Drafting, Singing, Mathematics, Discussions on Natural Sciences, and The Law of God (Religious Studies) (Tolstoy, 2006b, p. 29), was a result of the organic process coming from the students versus from the guiding power of the teachers and, especially, from Tolstoy. Based on its striking similarity to the structure of conventional schooling at his time and even now, I suspect the latter. I wonder if this conventional school structure, uncritically accepted by Tolstoy and, probably, by his other teachers, was imprinted on the Yasnaya Polyana school via “pervasive informality,” described by Jim Rietmulder, a co-founder of a democratic school in the USA:

Reflecting [on] the social movement [of the 1960s], many free schools sought to eliminate power, structure, and authority generally, sometimes tending not to distinguish the real targets of the revolution: abusive power, oppressive structure, and illegitimate authority. The result was not usually anarchy, but instead *pervasive informality*: informal power, informal structure, and informal authority. Some free schoolers tended to gloss over or “not see” the presence of power, structure, and authority in their schools. In contrast, the democratic schools in this book are about formal structure and formal authority — meaning structure and authority are visible, valued, acknowledged, and documented (Rietmulder, 2019, p. 15, italics mine).

I suspect Tolstoy’s similar blindness to his own power and the power of conventional institutions colonized his educational imagination (I will develop this point more later in this essay).

Nevertheless, I do not doubt that the emergence of the school order was a creative, evolutionary, and pragmatic process based on the freedom principle (and the pervasive informality). Also, I think Tolstoy might respond to my charge above by arguing that it might not matter from what structure to start a democratic school – it would evolve in the right direction under the principle of freedom from coercion and pedagogical pragmatic experimentation.

In a conventional school, a teacher’s lesson is an assignment, while I claim that the Yasnaya Polyana school teachers’ lessons were educational offerings. What makes the difference between an assignment and an offering is the students’ right to leave the teachers’ lessons at any time. Educational offerings provided by Tolstoy and his teachers to the Yasnaya Polyana school students could take diverse forms like reading books of the students’ choice taken from the school library; attending a teacher’s guided lesson; reading a book; telling a story; demonstrating a natural science experiment; working on a teacher’s assignment; dialoguing about the presented lesson; going to a nature fieldtrip (e.g., to wild woods); telling each other stories; teaching each other; and so on. There were no orderly arranged benches or desks in the classrooms. Each student had a right to leave a classroom or a school at any time. Tolstoy commented that it was often unpleasant for the teachers when students left their classrooms or school before the lesson time was over, but, he argued, that the alternative of using coercion at the expense of the sensitivity of guidance was much worse (Tolstoy, 1899b, pp. 184–185). There was freedom of movement and communication in the classroom. Some lessons or discipline subject matter were modified by the teachers on students’ demand: instead of teaching one subject, the teacher had to teach another one, or instead of teaching it for one period, it was taught for three. Students could also subvert the teacher’s learning activity without any repercussions. For example, Tolstoy reported that in a grammar lesson, children might move from quizzing each other on, “Where is the predicate?” to playfully giving each other puzzles like, “What sits in the spoon, letting his legs hang down?”, entirely subverting the grammar lesson to their entertainment (Tolstoy, 1899b, p. 223).

Not all educational offerings designed by the teachers were accepted by the students. For instance, the Yasnaya Polyana students found learning grammar boring and irrelevant. They could not understand what grammar was for. According to Tolstoy, the students felt that grammar was formalistic, deadly, meaningless, and oppressive. Tolstoy could not respond satisfactorily to the students’ questions about what grammar was for, for them or himself. He noticed that knowing grammar helped neither the children nor him to write as a professional literary writer. Also, he observed that the local dialect used a different grammar than the dialect of Russian schooled people: for example, the peasant local dialect used only two genders for nouns (female and male), while the dialect of Russian schooled people used three genders for nouns (female, male, and neutral). Sensing the children’s and his own hopeless alienation from grammar, Tolstoy considered dropping this subject offering altogether as he could not articulate the usefulness of learning grammar. Later, he developed similar doubts about the relevancy of teaching peasant children history and geography after many unsuccessful pedagogical experimentations with teaching these subjects.

Tolstoy was dissatisfied with the conventional teaching of history in Russian schools and elsewhere, focusing mostly on memorizing the dates of wars and other pompous historical events of people in power. He noticed that students found it tedious, boring, and irrelevant. His first innovative approach to teaching history was focused on exposing his peasant children to historical *narratives*, to the story part of history. He got this idea from his very successful teaching of the Old Testament, which so excited the peasant children’s imagination, creativity, and interest that the children enthusiastically shared not only with each other but also with their parents. Tolstoy reasoned that the Old Testament was nothing more than teaching the history and geography of the Ancient Jews. Why not apply the same approach to teaching the World and Russian histories?! One just had to develop literary stories involving other peoples and other geographies. To teach ancient histories and geographies of Ancient Egypt and Ancient Middle East, Tolstoy decided to exploit the Old Testament.

The result was ambivalent. Tolstoy was very successful in lively engaging his peasant students in the studies – the children were very willing to study the biblical stories on ancient history and geography he developed for them. But, according to his own observations and reflections, he failed in two important aspects. First, while studying the biblical stories, the peasant children focused on the religious aspects of the stories rather than on history or geography.

Second, their sympathies were always sided with ancient Jews, as God-chosen people, “ours” – our team to be a fan of. They had little interest in ancient Egyptians or Phoenicians.

Initially, Tolstoy attributed these two important failures to a lack of good and relevant historical-geographical literature for peasant children. When Tolstoy introduced such literature, like Semiramis, which the children liked, he noticed that the children “found something in ancient history to remember and enjoy, about Semiramis, for instance, but it was remembered merely accidentally, not because it cleared up anything, but because it was artistically related” (Tolstoy, 1899b, p. 257). In other words, the children’s interest was based on their aesthetic fascination with fictional literature rather than with history.

Tolstoy decided to change his approach to teaching history by focusing on (1) the recent history and (2) the local history of the motherland. He hoped that this relevance and familiarity would kindle the peasant children’s interest in history as such. He and his teachers tried to use the Socratic dialogic method of asking students questions, which initially looked promising, but then Tolstoy recognized its manipulative nature of forcing the students to say what the teacher wanted to hear (cf. Matusov, 2009, ch. 3). Then he switched to telling stories about the French invasion of the Russian Empire in 1812 and the more recent Crimea War campaign (1853–1856), in which Tolstoy himself participated as an artillery officer and journalist. These stories strongly impressed the peasant students through their deep sympathy for the Russian side in those dramatic wars: “I obtained the greatest success, as I might have expected, from my story of the war with Napoleon. This lesson made a memorable hour in our lives. I shall never forget it” (Tolstoy, 1899b, pp. 268–269). It looked like Tolstoy found his Holy Grail of teaching peasant children history.

However, soon, Tolstoy realized it turned out to be another pedagogical illusion of his. A visit of a German colleague who attended the most successful history lesson of the 1812 war sobered up Tolstoy. Tolstoy realized that his pedagogical success was not about teaching Russian history but Russian chauvinism: “I entirely agreed with him [Tolstoy’s German colleague criticizing the history lesson] that my narrative was not history, but a tale kindling the national sentiment. Of course, *as instruction in history* this [pedagogical] experiment also was even more unsuccessful than the first” (Tolstoy, 1899b, p. 272, italics original). The offering of history failed in the Yasnaya Polyana school. Tolstoy raised the question of whether authentic teaching of history to peasant children was possible or even necessary.

I want to make the following three observations on Tolstoy’s pedagogical experiments with teaching history to his peasant students. First, Tolstoy’s initial pedagogical belief that students’ lively engagement is the measure of the educational quality of teaching was an apparent mistake. Tolstoy might be right that students’ disengagement is a good measure of pedagogical failure, but the reverse was not necessarily true. Students can be highly and lively engaged in a lesson without learning something relevant to the teacher’s pedagogical desire. For example, Tolstoy’s students might learn the aesthetics of fictional literature involving historical narratives rather than history. Even more, this highly lively, engaged learning can sometimes be very harmful, like, for instance, enthusiastically learning about Russian chauvinism. I could not find any reflective discussions about that in Tolstoy’s writings, although I do not preclude his growing awareness of this fact based on his pedagogical experimentation.

Second, it was Tolstoy, not his students (or even his teachers! ), who made the value judgments on how educational the students’ learning was and why. Elsewhere, I argued that what distinguishes education from learning is precisely the educatee’s judgments about how the experienced or expected learning is valuable for the educatee (Matusov, 2021b). I could find some discussions of this by the students in Tolstoy’s writing, like, for example, the students’ challenges of the teachers, including Tolstoy, about the usefulness (for them) of studying grammar. There might have been even more private or public discussions involving similar judgments about the value of learning at the Yasnaya Polyana school among the children and/or between the children about their parents that Tolstoy might not have been aware of or did not include in his pedagogical writings. However, Tolstoy himself did not discuss the difference between learning and education or who should be the legitimate and primary judge of the educational value of learning. Or, again, at least, I could not find such discussions in the historical records available to me. In any case, in my view, this aspect of Tolstoy’s Yasnaya Polyana school challenges the democratic nature of the school – i.e., how much Tolstoy still tried to impose his curriculum on his students – and its educational qualities – i.e., how much his school was about learning and not about education. Thus, although without pedagogical coercion, his school arguably still employed *paternalistic* education.

Third, I really admire Tolstoy’s reflections on his pedagogical experimentations, focusing on his honest portrayal of his pedagogical failures and not only successes. The dramatism of his reflections was based on his very enthusiastic descriptions of his (initial) pedagogical success, which, later, on a close look or further testing, turned out to be a pedagogical disaster, many of which remained unresolved for him. I think Tolstoy contributed an important and interesting genre of pedagogical critical reflection. This is very different from a strong fashion among educators only to report their pedagogical successes. Let me provide an example of Tolstoy’s pedagogical experimentation through the reflection loop using an example of his teaching reading.

Tolstoy keenly criticized conventional teaching: “*The teacher is always involuntarily impelled to select for himself the most convenient method of teaching. The more convenient this method is for the teacher*,* the more unsuitable it is for the [student]. That method is the only good one which renders the pupils contented*” (Tolstoy, 1899b, p. 205). In solving the reading learning paradox that reading a text requires the mechanics of its sounding, while learning mechanics of the text sounding requires the reader’s interest in the text, conventional school often chooses mechanical reading instruction over comprehension, which is currently called “phonics” (Smith, 1985). The opposite approach to teaching literacy focused on reading comprehension, the so-called “whole language approach” (Smith, 1985). Tolstoy and his teachers initially gravitated to the comprehensive reading, whole language, approach. However, compared with traditional schools, the Yasnaya Polyana school children did not learn as well (or as fast), which led some parents to take their children from the school and move them to private, paid, tutors or schools. But the deeper problem discussed by Tolstoy was that in his observations the whole language approach worked well only for a very small number of children. I will skip the drama of his pedagogical experimentations and reflections but turn directly to his “final” pragmatic solution.

Tolstoy decided to observe how children learn to read naturally. From his observations of the students in his school, he abstracted the following forms of engagement of the students in reading: (1) reading with the help of a teacher, a parent, or a more advanced peer – basically, another person reads the text of the reader’s interest; (2) solo reading for the student’s own interest and ability – it is “very popular, and everyone who learns to read fluently makes use of it. In this case, the pupil is given a book and is left wholly to himself to make out and comprehend what he pleases” (Tolstoy, 1899b, pp. 207–208); (3) reading a favorite text by learning it by heart, which works for many autodidact readers; (4) reading together with peers; and (5) reading with comprehension, discussing the text with others, — “that is, the reading of books with interest and comprehension growing ever more and more complicated” (Tolstoy, 1899b, p. 209). Further in his writings, Tolstoy also reported the sixth form when some (but not all! ) students came to a teacher or a more advanced peer to ask for mechanical reading instruction (i.e., phonics).

Tolstoy argued that the goal of the school is to legitimize and support all these forms of naturally occurring learning to read and let the students flexibly and freely choose these forms that worked best for them at the given moment while providing diverse reading materials for the students’ diverse interests in reading and texts: “Had it not been for this freedom and external disorder which to some people seems so strange and impossible, we should not only never have hit upon these five [actually, six] methods of learning to read, but moreover we should never have dared to employ and proportion them to the demands of the pupils, and consequently we should never have attained those brilliant results which we attained in reading during the last part of the time” (Tolstoy, 1899b, pp. 209–210). Essentially, this pragmatic and anarchistic approach overruled Tolstoy’s cherished (constructivist) ideas about pedagogy and the purpose of literacy. His literacy writing profession highly influenced his initial definition of the purpose of literacy instruction: “The problem of instruction in language consists, in our opinion, in directing the pupils in the comprehension of the contents of books written in the literary language. The knowledge of the literary language is indispensable because all good books are written in it” (Tolstoy, 1899b, p. 202). In general, Tolstoy defined the primary goal of education as creative enculturation rather than critical transcendence of the given culture (Cohen, 1981). Later, he seemed to recognize that it was up to his students to define the purpose of the reading learning. This demonstrates how Tolstoy’s pragmatism, anarchism, and naturalism democratized his pedagogical views.

## Dialogic Education at the Yasnaya Polyana School

*For me*, as a democratic *dialogic* educator, the most exciting evidence of the quality of education at the Yasnaya Polyana school was children-initiated inquiry-based discussions. Dialogic education is my pedagogical bias. When the Yasnaya Polyana school children asked their teachers why people sing, or why study grammar, or which particular wordings in their writing stories were good or bad, or why some (male) adults get drunk and cruel to children, women, and animals, and so on – i.e., the inquiries of their genuine interest, – often these inquiries sparked exciting critical dialogues, in which children developed their authorial judgments and tested their ideas with each other, their teachers, and Tolstoy. Using Bakhtin’s terminology, the critical dialogues are based on “the internally persuasive discourse” (Bakhtin, 1991), where persuasion is internal to the discourse rather than the external or internal authority (Matusov & von Duyke, 2010). In the internally persuasive discourse, consciousnesses with equal rights to be taken seriously encounter each other (Bakhtin, 1999, p. 6). In such critical discussions, the value of word by Lev Tolstoy, a famous Russian writer, a recent slaveowner of the peasant children and their parents, a powerful landlord and aristocrat (a close relative to the Tzarist family), a Count, the owner, financier, and headmaster of the Yasnaya Polyana school, etc. became equal to the words of Fedka, Pronka, or Semka – peasant children, recent slaves of Tolstoy. His students could put down Tolstoy’s opinions if they did not survive dialogic tests – his opinions did not have special weight simply because he expressed them. It was beautiful to read how much peasant children’s words puzzled Count Tolstoy, how much he did not know how to reply to them, how much he was puzzled by them, how much he learned from his peasant children and valued this learning, how much he genuinely admired their authorial and unique judgments – how much these dialogic encounters were eventful for him and the children. One of his reflective essays was titled, “Who should learn writing of whom; peasant children of us, or we of peasant children” (Tolstoy, 1899b, pp. 301–334), which was incredibly amazing to hear from the world-renowned writer.

Most of these critical dialogues initiated by the students emerged in long field trips in the wild woods or freely emerged in the school offerings, especially when Tolstoy and the other teachers were not busy with teaching their preplanned curriculum or focused on making students lively and engaged in what they wanted the students to study. Thus, the more the Yasnaya Polyana school was committed and had fidelity to freedom from coercion (and, I’d add, engagement manipulation, common to Progressive Education – see below), the more dialogic education emerged in the school. Finally, it is not by chance that the students’ value judgments about the teachers’ offerings and their own emergent learning appeared in these naturally occurring critical dialogues.

## Resolving Conflicts in the Yasnaya Polyana School

Tolstoy seemed to believe that intense violent interpersonal conflicts among children, especially among boys, were unavoidable, and it was better for the adults (schoolteachers) to stay away from them for several reasons (Tolstoy’s attitude is somewhat similar to Japanese early educators’ attitude; see Tobin et al., 1989; Tobin et al., 2009). First, adults tend to stop a fight before it resolves itself and before anger and negative emotions release themselves. Second, adults tend to impose unjust peace on the involved parties, which often makes them even more grudging, angry, and cruel with each other than before adult interference. Third, physical fights often occur in children’s public spaces where other children can help to regulate and mediate the intensity of the physical fights and their possible physical and moral damage. Fourth, the natural communal process of self-regulation generates new communal unwritten norms of the noble ways of the fight conduct for the children. Fifth, the peace established through this physical fighting without adult intervention is much more lasting and genuine than otherwise it would be.

Tolstoy described several cases of boy fights at the Yasnaya Polyana school (Tolstoy, 1899b, pp. 173–175). He argued that the Yasnaya Polyana school’s hands-off approach led to much better outcomes than in conventional schools or settings, including limiting serious fight injuries to a minimum,

But perhaps the teachers who have not had experience of such disorder or free order, will think that without disciplinary interference this disorder may take on physically injurious consequences; that they will break each other’s limbs or kill each other.

In the Yasnaya Polyana school last spring, there were only two cases of serious damage being done. One boy was pushed down from the steps, and cut his leg to the bone, the wound was healed in two weeks; the other had his cheek burned with blazing pitch, and he carried a scar for a fortnight.

Nothing ever happened, unless perhaps once a week someone cried, and that not from pain, but from vexation or shame. Of blows, bruises, bumps, except in the case of the two boys just mentioned, we cannot recall a single one during all the summer among thirty or forty pupils, though they were left entirely to their own guidance (Tolstoy, 1899b, p. 175).

No cases of prolonged bullying among children were described or discussed by Tolstoy either because bullying did not happen at the Yasnaya Polyana school, or because he was not aware of it, or because he was insensitive to it. He did describe several cases of unpopular kids at the school, but again, he did not feel that the problem was serious or required his attention, relying on children’s self-organization to address it. Thus, Tolstoy described a case of “idiot Petka,” who was ostracized by the rest of Yasnaya Polyana kids because, as the kids said about him, “What a strange fellow Petka is! If you strike him — and even the little fellows sometimes pick on him, — he shakes himself loose and runs away!” (Tolstoy, 1899b, p. 176). It is unclear to me what Tolstoy’s judgment of this peer ostracism was: did he approve or disapprove of it, and why? I suspect (based on the tone of his writing) that Tolstoy approved the peer ostracism because the boy violated the hegemonic norms of masculinity that Tolstoy might (uncritically) subscribe to. Of course, this is only my suspicion, which can be wrong.

The Yasnaya Polyana school’s primary principle was not to use any discipline. However, some teachers fell back to their old habits of conventional school occasionally, “I am convinced that a school ought not to interfere in affairs of discipline that belong only to the family: that a school ought not to have, and does not have, the right to grant rewards and punishments; that the best police and discipline of a school is gained by [entrusting] the pupils with full powers to learn and to behave as they please. I am convinced of this, notwithstanding the fact that the old customs of disciplinary schools are so strong that even in the Yasnaya Polyana school we occasionally departed from this principle” (Tolstoy, 1899b, p. 176). Interestingly, although Tolstoy described cases when Yasnaya Polyana school children apologized to teachers, the reverse was absent, including in the case Tolstoy started describing in the quote above. Also, there was no discussion of conflicts among the Yasnaya Polyana school teachers, even though Tolstoy mentioned his disagreement with at least two new teachers:

We have four instructors. Two are veterans, having already taught [for] two years in the school; they are accustomed to the pupils, to their work, and to the freedom and apparent lawlessness of the school.

Two of the teachers are new; both of them are recent graduates and lovers of outward propriety, of rules and bells and regulations and programs and the like, and are not wonted to the life of the school, as the first two are. What to the first seems reasonable, necessary, impossible to be otherwise, like the features on the face of a beloved though homely child, who has grown up under your very eyes, sometimes seems to the new teachers a sheer disorder (Tolstoy, 1899b, p. 165).

More severe cases of disruption of school peace involved incidents of theft. Tolstoy described a few instances of theft, when “a Leyden jar was taken from the physical laboratory, pencils several times were missing, and books also were missing” (Tolstoy, 1899b, p. 177). When the two boys were discovered stealing the school equipment, The Yasnaya Polyana teachers let the children define the punishment for these two thieves. After considering and then rejecting whipping the thieves, which was the most common internal punishment for theft by the Russian landlords and the Russian peasant communities at the time, the peasant children put a placard-ticketed THIEF of the two boys. It is too bad that Tolstoy did not describe the process of making a decision, but he commented,

This punishment, [we are] ashamed to say, had been proposed by ourselves^11^ once before, and the very lad who a year before had worn a placard inscribed LIAR, now of all others was the one to propose the placards for the thieves (Tolstoy, 1899b, p. 177).

We decided on the placards, and when one of the girls had embroidered them, all the scholars looked on with angry pleasure, and ridiculed the offenders. They proposed a still more severe punishment: “To take them to the village, and make an exhibition of them with the placards on during the holiday,” was their proposal.

The offenders wept (Tolstoy, 1899b, p. 178).

Despite the psychologically tortious punishment, stealing was repeated again by one of the same thieves. This made Tolstoy angry at the involved boy. He wanted to punish the boy even more severely. But then he became ashamed of his emotions and desire for more violence:

I felt almost angry against the thief.

But as I looked into the culprit’s face, which was more pale, wretched, passionate, and hard than ever, I seemed to see the face of a convict, and it suddenly appeared to me so wrong and odious, that I took off the stupid placard; I told him to go wherever he pleased, and I suddenly felt the conviction — felt it, not through my intellect, but in my whole being — that I had no right to punish this unhappy lad, and that it was not in my power to make of him what I and the dvornik’s^12^ son might like to make of him. I felt a conviction that there are soul-secrets hidden from us on which life, but not regulations and punishments, may act (Tolstoy, 1899b, p. 179).

I want to make the three following observations about this case. First, it is interesting to me how Tolstoy *unilaterally* made this decision of mercy. Unlike in the first case of theft, when the decision was made either solely by the children or in collaboration with the teachers, here, the decision was made entirely by Tolstoy. Such unilateral decisions were relatively common in his writings about the school as evidence of his paternalism and undemocratic power (see my discussion below).

Second, it is unclear to me how normative or descriptive these cases were for Tolstoy. For example, was his unilateral action of mercy an aberration from educational philosophy or a norm? Also, how did he judge this case against his overall faith in democratic informal self-organization, naturalness, and hands-off approach? Was it a deviation or his prioritization of some other principle – possibly, the principle of his personal responsibility that might have the legitimacy to interfere in the face of open injustice and the child’s suffering, i.e., his civil disobedience and his growing opposition to any violence on the Christian grounds (see Tolstoy, 1968) – at work here? Alternatively (or in addition), it can be another case of “pervasive informality,” discussed above, when one tries to live according to the “internally felt moral conscience” rather than according to the “externally [democratically] accepted rules or laws.”

Third, in his pedagogical writings, Tolstoy never critically examined his power at the Yasnaya Polyana school as a slaveowner, a Count, a high-ranking aristocrat with high connections with the top government of the Tsarist Russian Empire and the civil society, the (recent) owner of the slaves – both the involved parents and children at the Yasnaya Polyana, – the founder of the school, the financier of the school, the owner of the school, a famous Russian writer, a wealthy landlord, highly educated and knowledgeable, adult, male, and so on. He had these powers and influences over his students, his teachers, and his students’ parents. But I wished he also focused on both visible and tacit hierarchical relationships that he had with the members of *his* school, including the fact that he unilaterally closed the school when he had a nervous breakdown in 1862. He was the school (Blaisdell, 2000)^13^.

In his pedagogical writings about the Yasnaya Polyana school, Tolstoy often emphasized how much he was treated by the children and occasionally by their parents as “equal” to them,^14^ and I believe his evidence. It suggests that Tolstoy was sensitive to the issue of power – although he only focused on documenting power equality with his students (and parents) and did not try to explore his power inequality at the school and what is, probably even more important, its legitimacy. Why is that? I can only speculate.

I have several hypotheses. First is the phenomenon of persuasive informality common to educators who commit to “equal power,” briefly outlined above, that often leads to hiding an educator’s inequal power from themselves and others. As a democratic dialogic pedagogy, I argue that totalized power equality is both unachievable and undesirable (Matusov & Marjanovic-Shane, 2015). Rather, the issue of legitimacy and illegitimacy (e.g., abuse) of power inequality must be problematized in a given context. Second, Tolstoy was severely criticized by the Russian Tsarist government functionaries, conventional and progressive educators, intellectuals, the press, and so on, for how his innovative democratic education, which put him on the defensive and led to his sliding into conservativism. He noticed this trend by commenting, “…nothing is more demoralizing to the regular conduct of a [innovative democratic] school than to have visitors” because the visitors force the innovative school to become defensive, which may degrade it into undesired conservatism that uncritically smuggles power hierarchy (Tolstoy, 1899b, p. 165). Also, he had to compete with conventional private and religious schools and tutors for the hearts of the fathers of his students who decided to allow their children to attend his Yasnaya Polyana school. Third, despite his criticism of conventional education’s paternalism, Tolstoy was ultimately unwilling to entrust students — both children and adults — with defining education as what was good for them to learn. Why was he not trusting enough? I think he might be under huge cultural, social, political, and institutional pressures, being also colonized by conventional paternalizing education. Alternatively, he might see legitimate limitations of the school democratic governance and justification for his unilateral school guardianship in some important (moral? ) issues, as a form of his civil disobedience to injust democratic governance (Tolstoy, 1968).

## Tolstoy’s Educational Philosophies

I can abstract at least four educational philosophies in Tolstoy’s approach to his Yasnaya Polyana school.

### Conventionalism

The first is the conventional philosophy of educational paternalism through coercion. What is the evidence for that? The organization of the school was very conventional with its clearly marked age- and ability-based classes, teachers-designed schedules, teachers’ grade marks, Tolstoy-designed overall disciplinary curriculum, Tolstoy’s judgment of what was educational and what was not, the teacher-designed lessons, and so on. All these organizational elements were paternalistic in their nature, in my view. Paternalism involves limiting the liberties of others based on an assumption that the person in power knows better what is good for the less powerful others (Dworkin, 1972).

Also, Tolstoy unapologetically used pedagogical coercion to make sure that the students were engaged in his lessons even when they might dislike them or find these lessons boring and irrelevant. The verb “to coerce” (“заставлять” in Russian) was very common in Tolstoy’s writings about his Yasnaya Polyana pedagogy, which was often translated in English as “to make,” like, for example, “Not naming the parts of speech in a sentence, *I made the scholars write something down*, sometimes giving them a subject—that is, a proposition; and using questions I tried to make them amplify the proposition by introducing adjectives, new subjects, qualifying clauses, relatives, and complementary attributes.” (Tolstoy, 1899b, p. 224, italics mine). Google Translate translated the original Russian sentence^15^ a bit more accurately to English, in my judgment: “Without naming the parts of speech of the sentence, *I forced them to write something*, sometimes asking the subject, i.e., the subject, and with questions, I forced them to expand the sentence, inserting definitions, new predicates, subjects, circumstances, and additions.” Most of such pedagogical coercions by Tolstoy led to even more student disengagements, “They did acquire them [i.e., grammar rules], but it became a bore to them, and they in their heart of hearts asked themselves ‘Why?’ and I was obliged to ask myself the same question, and could find no answer” (Tolstoy, 1899b, p. 225).

To Tolstoy’s credit, when his pedagogical coercion led to students’ increased alienation from the imposed learning, he abandoned the coercion. This makes me think that Tolstoy’s use of pedagogical coercion was conditional and counting on a possible increase in students’ lively engagement and, thus, was expected to be temporary. Suppose my interpretation of Tolstoy’s possible justification of his pedagogical coercion is correct. In that case, it may raise the question of whether his pedagogical coercion is a birthmark of his philosophical conventionalism and paternalism or a specific version of his educational democratism, trying to be a benevolent dictator for the creation of new voluntary educational processes (see my recent paper on a teacher as a benevolent dictator in democratic educational settings, Matusov, 2023d).

In my analysis, the sources of Tolstoy’s educational conventionalism were rooted in his own conventional upbringing, both at homeschooling and at the university he attended, which often remained uncritically accepted by him. Also, he experienced external pressures from the government and cultured Russian civil society, constantly comparing the educational successes of his Yasnaya Polyana school students with a variety of conventional schools. Similarly, Tolstoy had to take into account the pressures for conventionalism from his students’ parents and especially fathers because, although school attendance was free, it was the fathers who chose to allow or disallow their children’s school attendance. In addition, some pressures for conventionalism (e.g., grade marks) came from the students themselves because they might be familiar with conventional private schooling for peasants and not only. Finally, the Yasnaya Polyana school existed for only three years – I wonder how much Tolstoy would have shaken off his conventionalism if his school had survived much longer.

Tolstoy’s overall commitment to students’ freedom from educational coercion and his faith in the organic emergence of self-organization might help him and the school more critically reflect on educational conventionalism and distance itself even more from it while pushing back diverse pressures for it. And Tolstoy was the first democratic educator for young students – he did not have much support for his revolutionary ideas rooted in the past. He was a pioneer of democratic education and could not have a like-minded community of democratic educators to consult with.

Finally, Tolstoy did not seem concerned with his own conventionalism, probably because of his anarcho-organic pedagogical approach: it does not matter how wrong the initial pedagogy is — it will be corrected by the coercion-free feedback loop.

### Progressive Education

The second educational philosophy I found in Tolstoy’s pedagogical writings was Progressive Education. Progressive Education also accepts educational paternalism but tries to realize it not so much via open coercion, but by manipulation of the student’s consciousness: “…let him always think he is master while you are really master” (Rousseau, 1979, p. 120). I noticed at least two major mutually related marks of Tolstoy’s Progressive Education. One is his focus on students’ high lively engagement as the measure of the quality of education. The other one is a teacher’s pedagogical efforts to fascinate his students with the subjects he was teaching:

If these teachers know how to make their lessons [fascinating^16^], these lessons will be useful in spite of their seeming incompatibility and accidentalness (Tolstoy & Blaisdell, 2000, p. 195).

We made various experiments in teaching grammar, and [I] must confess that no one of them succeeded in our aim of rendering this study [fascinating]. In the summer, in the second and first classes, a new teacher made a beginning with explaining the parts of speech, and the children – at least some of them at first – were interested, as they would have been in charades and enigmas. Often, after the lesson was finished, they recurred to the idea of enigmas, and amused themselves in puzzling one another with such questions as, ‘Where is the predicate?’ or.

‘What sits in the spoon,

Letting his legs hang down?’ (Tolstoy, 1899b, p. 223).

The child and the [adult] are receptive only in a condition of excitement; therefore to look on the joyous spirit of the school as something inimical is a brutal mistake which we too frequently make. But when this excitement in a large class becomes so violent as to prevent the teacher from managing his class, how then can you avoid shouting at the children and quenching this spirit?

If this excitement has study for its object, then nothing better could be desired. But if it be directed to some other object, then it is the teacher’s fault, since he does not regulate this spirit. The teacher’s problem, which is almost always solved unconsciously, consists in all the time providing food for this zeal and gradually getting it under control (Tolstoy, 1899b, p. 241).

No [adult] or child ever would have the power to study if the future of his teaching presented to him merely the art of writing, reading, or reckoning; no teacher could ever teach if he had not in his control views of the universe loftier than his pupils had. In order that *the pupil may wholly surrender himself to the teacher*, there must be opened before him one corner of that curtain which hides from him all the charm of that world of thought, knowledge, and poetry into which education is to lead him. Only when the pupil finds himself under the constant charm of this light gleaming before him will he be in a condition to work over himself as we require him to do….

I tried reading the Bible to them, and I completely conquered them. The corner of the curtain was lifted, and they gave themselves to me heart and soul. They began to love the book, and teaching, and me. All I had to do was to lead them on farther.

After the Old Testament I took up the New Testament; they loved learning and they loved me more and more (Tolstoy, 1899b, p. 251, italics mine).

Encouraging the children to play games like charades and enigmas was a pedagogical strategy to smuggle the grammar curriculum into those playful activities voluntarily chosen by the children. Progressive Education sees the goal of the teacher as constantly exciting and controlling the student’s lively engagement in the subject and topic targeted by the teacher to exploit the natural, often tacit, learning that emerges in this process. Recently, a similar idea of designing fascinating games that would smuggle academic learning was articulated by another Russian educationalist, Alexander Lobok (see his interview and our follow-up critique, Matusov et al., 2019, pp. 80–87, 175–198). This is a rather typical move by Progressive educators (Matusov, 2021a). Unfortunately for Tolstoy and his teachers, in the cited case above, the children moved quickly away from the grammar curriculum.

As I pointed out above, to Tolstoy’s credit, he dismissed student lively engagement as the marker of the quality of education, in contrast to many progressive educators. While he managed to engage his students in passionate discussions of historical texts or narratives, he realized that he was teaching, at best, literary aesthetics but, at worst, Russian chauvinism. Still, through his three-year experience of teaching and running the Yasnaya Polyana school, he remained committed to the educational paternalism of designing student curricula either through conventional or progressive means.

Another limitation to his Progressive Education that I observed in Tolstoy was that, unlike many Progressive educators, Tolstoy did not try to find the Holy Grail of a learning activity or pedagogical approach (Matusov et al., 2019) that, according to the motto of Progressive Education, “…any subject could be taught to any child at any age in some form that was honest” (Bruner, 1986, p. 129). As we can see above, Tolstoy questioned whether it was possible or even desirable to teach grammar, history, or geography to his peasant children at that time or at all.

Tolstoy was attracted to some of the Progressive Education aspects, like individualism, naturalism, liveliness^17^, high engagement, fascination, and attendance to the students’ subjectivities (i.e., child-centeredness), but disagreed with others, like forced collectivism, manipulation, and social conformism (Cohen, 1981).

### Democratic Education

Tolstoy’s Democratic Educational philosophy was evident and rooted in his conviction in student freedom from educational coercion and promoting the student’s inner freedom (Cohen, 1981), his trust in students (and people in general), respect for human dignity, his belief in the existential need for education in all people, his high value of self-actualization, and his beliefs in naturalism and organicism. According to the naturalist paradigm, people are good by their nature – when left to their own devices, they are “*naturally* inclined to learn and grow in healthy ways” (Miller, 2002, p. 63, italics original), or as Rousseau (1979, p. 92) put it, “the first impulses of nature are always right; there is no original sin in the human heart.” The latter probably involved his anarchist convictions that the natural development of things will always lead to the emergence of good organic self-organized structure from the initial chaos. Organicism is a belief in the self-organizing, self-reproducing, responsive, and evolutionary process, united in an organic whole, as one that brings and ensures good in contrast to the mechanically organized, pre-designed, usually hierarchical process, which is often viewed by the proponents of the organicism as “artificial and phony” (Goodman, 1971; Miller, 2002). Like Rousseau, Tolstoy seemed to believe that when left to their own devices, people naturally, or at least eventually, are good and that all evil comes from mechanically organized society – its culture, institutions, state, practices, violence/coercion, and values. Tolstoy apparently saw his Yasnaya Polyana democratic-dialogic school as a remedy for societal corruption. Yet, he observed that when he let the children solve their interpersonal problems, they often developed very cruel psychological punishments for their peers’ transgressions – that fact apparently remained unanalyzed by Tolstoy in his writings.

Tolstoy’s trust in his students – both children and adults – as authors of their lives was admirable. Even when Tolstoy disagreed with how his students defined their education, he followed their wishes, – for me this pedagogical feature constitutes the birthmark of a democratic educator (Matusov, 2020). For example, he noticed that most of his few adult students at the Yasnaya Polyana school approached their education instrumentally, in a very narrowly utilitarian way. Thus, according to Tolstoy, they tried to shorten time by intensifying their learning of how to read and write to the point that it became such a mechanical drilling that it was counter-productive to them. And yet, he accepted and supported their goals and methods. He articulated his understanding of their conditions – being ashamed to study with little children, taking time from their work and families, etc. – and sympathized with them while criticizing this type of education (Tolstoy, 1899b, pp. 197–198). In this way, Tolstoy accepted that education might be very pragmatic, instrumental, and even technological for his adult students and not necessarily intrinisic and existential, as he argued in his manifesto essay discussed above. Also, Tolstoy questioned the effectiveness of these adult students’ learning, and yet he did not try to take over it.

### Dialogic Education

Finally, I argue that Tolstoy subscribed to the Dialogic Education philosophy. This subscription was “in action” (Argyris & Schön, 1978; Latour, 1987) rather than “espoused” (Argyris & Schön, 1978). This means that Tolstoy practiced Dialogic Pedagogy rather than described or discussed it. He was susceptible to and interested in any child’s genuine interest, inquiry, puzzlement, and curiosity, and he was seriously supported by his mind and heart. At the same time, he apparently did not allow his own inquiries and interests to take over the children’s.

## Conclusions

In conclusion, I want to return to the three main questions that I raised about Tolstoy’s educational practices and philosophy.

1. Was Tolstoy a democratic educator in his theoretical ideas and his practice at the Yasnaya Polyana school? If so, what was the evidence of that?

My answer is yes, Tolstoy was a democratic educator. Why do I think this way? Primarily, it is because Tolstoy rejected the Enlightenment belief that education must be coercive in its nature. In his programmatic 6-page essay, “What is Enlightenment?”, the German philosopher Kant (1784) argued that on the way to promoting human dignity, understood as autonomy – the highest human value – there are two major obstacles. The first obstacle is the external tutelage, which can be addressed politically (e.g., revolution, reforms). The second obstacle is self-inflicted tutelage, which can be remedied only by compulsory education. Ignorant, immature, ill-informed, ill-intended, irrational people cannot be trusted with their autonomy – they must first be forcefully educated. Kant’s disciple Fichte formulated this point succinctly in the following way with the ultimate clarity: “Compulsion is also a kind of education” (cited in Berlin & Hardy, 2002, electronic edition). In his program essay, “On People’s Education,” Tolstoy rejected Kantian educational paternalism.

In my view, Tolstoy offered several major objections to Kantian educational paternalism. First, Tolstoy rejected the segregation of people into those who deserve dignity and those who do not – whose dignity has to be postponed and earned (Matusov, 2024). Second, Tolstoy rejected Kant’s premise that reasonable, educated people know better than the rest what the rest need to study or learn. Third, Tolstoy claimed that education is a universal existential need and desire and, as such, cannot be and must not be forced. Fourth, educational coercion makes education and guidance insensitive to the educatee’s interests and needs because it breaks the feedback loop on which such sensitivity can only be based. When something does not work in an educational practice, which often leads to the educatee’s alienation and disengagement, the designers of coercive education often focus on disciplining or manipulating the student into submission rather than on revision of educational goals, educational philosophy, curriculum, instruction, and so on.

Tolstoy was not an unconditional opponent of coercion in education. Instead, he was willing to use educational coercion in a very limited way to jump-start intrinsic educational processes in his students, which do not require continuing coercion afterward. When his coercive attempts failed, and usually they did, according to his account, he stopped his coercive efforts. Basically, through pedagogical coercion, he wanted to expose his students to some educational experiences that could induce new educational desires. For his pedagogical coercion, Tolstoy used the credit of trust that he earned at his students by being relevant, useful, helpful, and meaningful in the past. He indirectly told them, “Bear with me for a while. I will limit your freedom and impose some pains, but then you might highly appreciate my coercion by experiencing exciting, meaningful, fascinating, interesting, and important (for you! ) learning activities.” When Tolstoy failed the peasant children’s expectations many times in a row, he might have started losing his students who decided not to come to his lessons or entirely to his school. I think he knew that well and was, therefore, very careful and measured how often and intensely he could use pedagogical coercion.

Elsewhere, I wrote, “The litmus test for democratic education is for the student to be able to overrule the teacher (when the student invites the teacher in the first place, of course)” (Matusov, 2022, p. 24). I think Tolstoy passed this test. His students legitimately overruled his definition of language art education and hijacked many of his learning activities, turning grammar studies into playing charades, studies of history into aesthetic activities of storytelling or Russian chauvinism, and so on. The students could leave the lessons or the school at any time due to their dissatisfaction. Tolstoy’s adult students redefined the purpose of their education narrowly instrumentally, and Tolstoy accepted it, although remaining critical and disagreeing.

2. What kind of democratic educator was he? What democratic education practices and democratic education philosophy did he subscribe to?

In my judgment, Tolstoy’s democratic education was based on educational offerings to the students that he designed through his pedagogical experimentations, reflections, and revisions. At his time, Tolstoy conceptualized these offerings in terms of conventional academic disciplines common in conventional institutionalized and home education. Students could accept, reject, redefine, or even hijack these educational offerings. Students could accept an offering when engaged in it in the ways designed and expected by Tolstoy. Students could reject an offering by walking away from the lesson or school: “On the door of his school, Tolstoy hung a sign ‘Enter and Leave Freely’” (Cohen, 1981, p. 243). Students could redefine an offering by transforming it into another learning or non-learning activity, including recreational activity, which was not necessarily in line with the learning activity predesigned, expected, or approved by Tolstoy. Students could hijack an offering when they transitioned to a completely different activity, unrelated to the fare defined by Tolstoy and the other teachers at the Yasnaya Polyana school.

In other words, Tolstoy affirmed education’s authorial nature for both the educatees and educators (Matusov, 2011, 2020). Authorial education means that the educatees and educators participate in their educational practices with their “minds and hearts” (Matusov et al., 2019): their personal convictions, personal opinions, personal beliefs, personal curiosities, personal interests, personal creativity, personal needs, personal feelings, personal imagination, personal necessities, personal concerns, personal dreams, personal goals, personal sensibilities, and so on. The notion of authorial education is contrasted with the notion of instrumental education when teachers often try to instill the “correct” ideas, beliefs, and skills in the students. Consequently, students often try to guess and produce what the teachers want from them. Tolstoy referred to the latter using the Russian term “vospitanie” (“воспитание”^18^), which he associated with coercion and manipulation.

I suspect that Tolstoy was an *educational dualist*, as my colleague Ana Marjanovic-Shane and I described it (Matusov & Marjanovic-Shane, 2016). On the one hand, he was an educational visionary, a proponent of a particular goal of education, such as creative enculturation. On the other hand, he was a radical educational pluralist, prioritizing an educator’s vision of education, which can be different from his own even if he disagrees with such a vision.

As to interpersonal conflicts, Tolstoy either argued for adults to stay away from them in cases of children’s fights or delegated the students to address more severe problems disrupting the school peace, such as theft. However, at times, Tolstoy seemed to feel the necessity to interfere and overrule the students’ collective punishment in some situations when he apparently felt his strong moral convictions went against the children’s democratic decisions.

Philosophically, in my judgment, he was committed to educational anarchism, naturalism, self-organization, feedback loop, non-coercion, and dialogism (the latter was in his pedagogical practice but apparently not conceptually).

3. What were the limitations of Tolstoy’s democratic education? What were the reasons for those limitations?

I think that the primary limitation of Tolstoy’s democratic education was his non-recognition and his tepid support of the student’s educational agency and, specifically, its four most important aspects: (1) it is up to the student to define what is educational for them and what is not, (2) it is up to the student to decide not to participate in education, (3) the student’s educational activism in designing their own learning and non-learning activities, and (4) the students’ organization of their own life in the school. I argue that Tolstoy monopolized these four aspects of the student’s educational agency. To his credit, when these four aspects spontaneously emerged in the students, Tolstoy did not actively suppress them: when a new learning activity emerged, Tolstoy did not block it; the students had the right to leave the classrooms and the school; when the students questioned or redefined the purpose of their education, Tolstoy listened to them attentively, and if they insisted, followed their definitions; and Tolstoy tried to stay away from the students’ life outside of the classrooms. Nevertheless, he definitely used the weight of his authority and persuasion to try to channel the students’ educational agency in his direction. Only when his persuasion and authority failed did he give up and let the students’ agency rule. I could not find in Tolstoy’s writings his attempts to diversify legitimate options for his students, like possibilities to play or engage in diverse hobbies and activities in parallel to the offered classes, non-lesson-based offerings (in addition to fieldtrips), students’ self-organization, possibilities for clubs, and so on. Tolstoy’s democratic school was mainly, if not excessively, based on his benevolent dictatorship (cf. Matusov, 2023d) not only with regard to his students but also his colleagues and teachers at the Yasnaya Polyana school, although probably in a different way.

Tolstoy defined the purpose of education as the transmission of knowledge and skills from the teacher to the student: “…the activity of education comes to a close immediately upon having reached a point of equality in knowledge [between the student and the teacher]” (Tolstoy, 1967, p. 185). In this “self-evident truth,” as he put it, he remained colonized by the conventional educational philosophy. He also implicitly recognized the right of the educators to impose the definition of education (i.e., what learning is good for the educatee) and its purpose for the educatee, which was realized in the nature of the educational offerings designed by the teachers. Interestingly, we can find the opposite idea in Tolstoy’s writings, criticizing teachers for making students think as they think (see his quote about pedagogical violence above). I can conclude Tolstoy’s insistence on educational non-coercion, which he practiced as an overall principle, did not save him from educational paternalism. Thus, Tolstoy’s Yasnaya Polyana school demonstrated that non-coercive, non-foisted education is not necessarily equal to non-paternalistic education. I wonder if his pedagogical experiment in Democratic Education ran long enough, his non-foisted education might progress into non-paternalistic education.

Of course, because of his pedagogical pragmatism and anarchism, such contradictions were an inherent part of his educational philosophy and practice of self-improvement through the teachers’ experimentation, non-coercion, students’ feedback on the teachers’ offerings (by voting with their feet, hijacking these offerings, and modifying them), and educational self-reflections. Tolstoy rejected conceptual coherence, any pedagogical system, any abstract universal (decontextualized) pedagogical theory or method in his fidelity to pedagogical pragmatism, heuristics, anarchism, freedom from coercion, and naturalism. Archambault (1967, p. iv) characterized Tolstoy’s pedagogical views as being “anti-theoretical.” Tolstoy saw teaching as an art and not as a method; teaching “is not a method, but an art and talent” (Tolstoy & Blaisdell, 2000, p. 187).

I think that Tolstoy overemphasized the role of pedagogy in education. Although he acknowledged autodidacticism, peer learning clubs, and self-directed education here and there in his writings (e.g., Tolstoy, 1967, p. 135)^19^, he often defined education through the relationship between the educator and the educatee – even more, through a pedagogical action of the educator on the educatee, “…by the word school, I mean, in the most general sense, *the conscious activity of the educator on the educatee*, that is, one part of education, no matter how it is expressed this activity: teaching the military order of recruits is school, reading public lectures is a school, teaching a course in Mohammedan madrasa is a school, museum collection and opening it for those who wish to attend is also a school” (Tolstoy, 2006a, p. 241, italics original)^20^. In general, I think Tolstoy was highly influenced by Rousseau’s progressive education to the point he could not shake it off, although he also tried to overcome this influence (Archambault, 1967). I think Tolstoy had a love-hate attitude to Rousseau. Tolstoy probably sensed a totalitarian tendency in Rousseau, especially in his “The Social Contract,” which later was fully explored by Isaiah Berlin (2002).

Tolstoy either did not know or did not appreciate the Ancient Greek notion of “school,” which literally meant “a type of leisure”^21^ among other types of leisure such as play, hobbies, and hanging out with friends (Matusov, 2020). Instead, Tolstoy purified education from other activities and, especially, from other forms of leisure, as separate and purposeful practice, which contradicted his own claim about unconscious education of everyday life (Tolstoy, 1967). Most modern democratic schools (e.g., Greenberg, 1991; Neill, 1960; Rietmulder, 2019) recognize that: (1) education might take different forms, including leisurely ones, (2) education is not purified from but embedded in many other leisurely and non-leisurely activities of and by children, (3) both deliberate (“conscious,” in Tolstoy’s terms) and non-deliberate (“unconscious”) education is recognized and supported, and (4) school democratic self-organization is supported and promoted.

I think there were many reasons and sources for such limitations of Tolstoy’s Democratic Education. The first source was conventional and progressive hegemonic education colonizing him – this colonization was not always visible to him or reflected by him, in my view. The second source was rooted in diverse pressures that Tolstoy experienced from the Russian Tsarist government, visitors, the general educated public, parents, the local peasantry, and even from his students who either criticized or actively undermined his pedagogical practice, making Tolstoy and his colleagues defensive and conservative at times. The third source was his unreflected powers and their (il)legitimacy both conceptually (i.e., espoused) and in-practice that directly or indirectly interfered with his democratic education project. His unreflected powers often manifested themselves through pervasive informality rooted in his political, religious, and pedagogical anarchism. Finally, he was professionally rather lonely, like many pioneers.

### Tolstoy’s Influence on Democratic Education

I see the two following major successors of Tolstoy’s Yasnaya Polyana school, the first democratic school for children, and his pedagogical conceptual writings. The first is the George Junior Republic^22^ movements in the USA (1890-present, see George, 1910). The second is the international democratic school movement, started by the Summerhill school^23^ in the UK (1921-present, see Neill, 1960) and the Sudbury Valley School^24^ in the USA (1968-present, see Greenberg, 1991). So far, I could not find any direct influences of Tolstoy’s democratic education on Summerhill, but a co-founder of the Sudbury Valley School, Daniel Greenberg, used a quote from Tolstoy in an epigraph to his book (Greenberg, 1991):

What is meant by non-interference of the school in learning? … [It means] granting students the full freedom to avail themselves of teaching that answers their needs. and that they want. only to the extent that they need and want it; and it means not forcing them to learn what they do not need or want. ….

I doubt whether [the kind of school I am discussing] will become common for another century. It is not likely … that schools based on students’ freedom of choice will be established even a hundred years from now (Tolstoy, 1967).

A scholar of Tolstoy’s educational philosophy claims that “Some of the best educators in our generation, among the advocates of education, such as Paul Goodman, John Holt, Edgar Freidenberg, George Dennison, and others, absorbed many of Tolstoy’s ideas, and used them as their own educational philosophy” (Cohen, 1981, p. 241), – but this claim has to be studied further. However, I agree with the following claim in Wikipedia about Lev Tolstoy “…as a direct forerunner to A.S. Neill’s Summerhill School [and, I would add, William George’s Junior Republic], the school at Yasnaya Polyana can justifiably be claimed the first example of a coherent theory of democratic education.” 25

## Data Availability

No datasets were generated or analysed during the current study.

## Footnotes

1 Aka Leo Tolstoy (1828–1910) was a famous Russian writer, the author of the renowned novels “War and Peace” and “Anna Karenina,” an educator, religious and political leader who coined the notion of “non-resistance,” see https://en.wikipedia.org/wiki/Leo_Tolstoy.

2 Tolstoy began his educational experiments at Yasnaya Polyana around 1849 (Tolstoy, 1967, p. 86), initially with informal teaching activities. In preparation for his pedagogical experiment, Tolstoy traveled to Germany, France, and England to observe the most innovative and advanced pedagogical practices and theories, which he criticized and rejected later on: “His visits in the European schools gave Tolstoy a gloomy picture of the sterility of the subjects, and the lack of vitality or imagination in the teaching method” (Cohen, 1981, p. 242). The school was formally established in 1859, coinciding with Tolstoy’s deepening interest in educational theory and practice. The school’s most active and influential period was in the 1860s. During this decade, Tolstoy was deeply involved in educational activities, developing unique teaching methods and curricula. The school did not operate continuously. It experienced periods of closure and revival, largely dependent on Tolstoy’s presence at Yasnaya Polyana and his other commitments. The exact year of the final closure of the school is not precisely documented, but it is generally believed to have been in the late 1870s or early 1880s. Tolstoy’s increasing preoccupation with religious, civic, and philosophical issues led to a shift in his focus away from educational endeavors (Tolstoy & Blaisdell, 2000). Soviet scholar of Tolstoy, Viktor Shklovsky, speculated that Tolstoy’s pedagogy was heavily influenced by Cossack childrearing: “Tolstoy sees in the Cossack community a model for all of Russia—peasantry without the nobility. This interpretation applies both to the period of completing [the novel] ‘The Cossacks’ and to the time of Tolstoy’s work in the Yasnaya Polyana school. The Yasnaya Polyana schools, with their free attendance and departure, without a defined [curricular] program, founded for the discovery and cultivation of talented children, are like would-be Cossack schools, as if continuing the ideas of Cossack society in pedagogy. This gang of children without bosses is the realization of the dream about Cossack society” (Shklovsky, 1963, pp. 140–141, translation from Russian is mine). Cossacks were free peasants who escaped serfdom and lived in specially designated land. However, neither Tolstoy nor other scholars, besides Shklovsky, mentioned the Cossack influence on Tolstoy (Blaisdell, 2000, pp. 7–8, 13).

3 In Russian “О народном образовании». It was translated into English as “On popular education” (Tolstoy, 1967), but I prefer “On people’s education” because in his manifesto, Tolstoy argued for the education of people, for people, and by people, at least in my interpretation. In this article, I used both Russian original texts and English translations of some of them.

4 I’m using the “coercion” translation of the Russian term “принуждение» rather than “compulsion,” the term commonly used in English translations (e.g., Tolstoy, 1967) because coercion is a broader term than compulsion. Also, “compulsory education” is translated as “обязательное образование» in Russian (literally, “obligatory education”). For example, Tolstoy included university education as an example of coercive education while, strictly speaking, it is not compulsory (yet? ) because people have the right not to attend universities, “Public lectures and museums are the best examples of [noncoercion] in education. Universities are examples of [coercion] in education. In universities, the students are confined to certain limits by a definite course, a program, a code of selected studies, by the exigencies of the examinations, and by the granting of rights based mainly on these examinations or, more correctly, by the stripping of rights in case of noncompliance with certain prescribed conditions…” (Tolstoy & Blaisdell, 2000, p. 193). In my own writing, I also use the terms “foisted education” and “paternalistic education” to broaden even more this term and include manipulation rather than only force, violence, the threat of violence, or imposition (Matusov, 2021b, 2022).

5 The use of the male pronoun here is not evidence of Tolstoy’s sexism but a part of the Russian language grammar where often (but not always) gendered pronouns refer to the gender of the corresponding noun (i.e., “child,” “rebyonok,” ребёнок, in Russian, which is grammatically a male noun).

6 In English historical literature, the term “serfs” is usually used to describe Russian “крепостные крестьяне.” Like many Russian historians, I prefer the term “slaves” as there was not much difference between slaves and “крепостные крестьяне.” Like slaves, “крепостные крестьяне” could be sold as a family or separately (with or without land) and could be beaten, tortured, raped, or even killed. “Крепостные крестьяне” must work for free and pay taxes to their owners (Gogolʹ, 2004). Finally, both Russian крепостные крестьяне and their owners commonly used the term “slaves” (“рабы») in Russian to describe this bond. There were three types of peasant slaves in Russia: private, state, and church owned. The majority of the “крепостные крестьяне” were ethnically Russians. However, some Russian peasants were free, https://en.wikipedia.org/wiki/Serfdom_in_Russia.

7 https://community.middlebury.edu/~beyer/courses/RUSS0101a-f05/Tolstoy/aboutme.htm.

8 Tolstoy used a quote from the Bible, Matthew 18:20: “For where two or three are gathered together in *My* name, *I* am there among them” (The New Revised Standard Version).

9 The motto is attributed to Pierre-Joseph Proudhon, a French theoretician of political anarchism. Tolstoy’s educational anarchism was very different from many other anarchist efforts in education that blossomed after Tolstoy because the latter (e.g., Ferrer Guardia, 1913) focused on *education for anarchism* – i.e., how to raise (“воспитать” – see below on “воспитание”) good adult anarchists (see Haworth, 2012, for more discussion), – while Tolstoy focused on *anarchistic education* – i.e., the emergence of educational self-organization from a chaos (cf. Feyerabend, 1975).

10 Tolstoy commented that using grade marks was a dying out pedagogical practice, “a relic of a past system” (Tolstoy, 1899b, pp. 180–181) – but still, why was it there at the Yasnaya Polyana school in the first place?

11 In the original Russian text, it is much clearer that Tolstoy alluded that the teachers, including Tolstoy himself, introduced this psychological punishment of shaming the morally transgressed children to replace severe physical punishment, which was so familiar to the peasant, former slave, children. I suspect Tolstoy realized here that this psychological punishment might represent much more torture to its victims than the traditional corporal punishment of whipping the thief (cf. Foucault, 1995).

12 A *dvornik* back then was a man who took care of the yard, landscape, and the pavement in the front of the slaveowner’s mansion.

13 Mark Smith commented on this point, “Reminds me a bit of the charismatic influence of the Neills (father, mother, and daughter) on Summerhill [democratic school]. Appleton (2000), I recall, described in his book how Zoe [Neill, the daughter of the school founder who became the head of the school] would come into the school once or twice per week and kind of energetically ‘hover’ over the school in some ways when she wasn’t there.”

14 For example, sometimes, during the intense collaboration on their writings, some peasant children used the familiar pronoun “ty” instead of the polite, formal, and respectful “Vy” to address the Count, landlord, teacher, schoolmaster, and adult Tolstoy, who never corrected them.

15 “Не называя частей речи предложения, *я заставлял их писать что-нибудь*, иногда задавая предмет, т. е. подлежащее, и вопросами заставлял их расширять предложение, вставляя определения, новые сказуемые, подлежащие, обстоятельства и дополнения» (Tolstoy, 2006b, p. 68, italics mine).

16 I think “fascinating” is a better translation of the Russian word “занимательный” than “interesting” – the original translation in this quote, or “attractive” in the following quote.

17 I wonder how much Tolstoy’s insistence on the liveliness of students is similar and different from the later notion of authenticity in education (see, for example, the discussion of the free school movement in Miller, 2002). Was Tolstoy’s term liveliness an external manifestation of authenticity? Or, it was the reverse: the liveliness as the expression of the person fully being here-and-now was a rejection of such thing as the authenticity of the self – the notion that emerged much later in a middle-class culture in the 20th century (Matusov & Smith, 2012).

18 This term is absent in English. I’d define it as “forming a good breeding with regard to a person (often a child) through forming their values, beliefs, habits, tastes, manners, and desires.” This Russian term was mistranslated as “culture” in English publications (see, for example, Spring, 2008, pp. 88–90; Tolstoy, 1967, pp. 105–151). See also the German terms *Klugheit* and *Civilisierung*, used by Kant and translated as “cultural refinement” and defined as being pleasant for others in manners and tastes to use others for one’s own ends: “The latter [i.e., *Civilisierung*] requires manners, courtesy, and a kind of discretion which will enable him to use all men for his own ends. This refinement changes according to the ever-changing tastes of different ages” (Kant, 1803/2012, #18). The Russian term воспитание embraces that as well.

19 Elsewhere, I abstracted at least five major types of self-education: (1) autodidact (solo study), (2) symdidact (study with peers), (3) autodidact with advisement, (4) odigόsdidact (study under the guidance of the teacher, but controlled by the educatee), and (5) autopaternalism (when the educatee asks an educator to force them to study what the educatee wants to study) (Matusov, 2022). Out of the five major types of self-education, Tolstoy mainly focused on one, namely odigόsdidactic education.

20 This is my translation from Russian to English with the help of Google Translate. I am not satisfied with the existing English translation, “By the word ‘school’ I understand not the house in which the instruction is given, not the teachers, not the pupils, not a certain tendency of instruction, but, in the general sense, *the conscious activity of him who gives culture upon those who receive it*, that is, one part of culture, in whatever way this activity may find its expression: the teaching of the regulations to a recruit is a school; public lectures are a school; a course in a Mohammedan institution of learning is a school; the collections of a museum and free access to them for those who wish to see them are a school” (Tolstoy, 1967, p. 143, italics is original).

21 Ancient Greeks viewed school as a form of leisure only for “free people,” whom they defined as ones who mostly were not governed by the necessities of life. Thus, they did not consider dependent children, including children of free people (mostly aristocratic men) free people. In their views, schools, as leisurely education, were not for children. It is interesting that Tolstoy defined such education of self-actualization for his peasant children (see, for example, Tolstoy, 1899b, pp. 195–196).

22 https://en.wikipedia.org/wiki/George_Junior_Republic.

23 https://en.wikipedia.org/wiki/Summerhill_School.

24 https://en.wikipedia.org/wiki/Sudbury_Valley_School.

25 https://en.wikipedia.org/wiki/Leo_Tolstoy.
